# Open-Porous Hydroxyapatite Scaffolds for Three-Dimensional Culture of Human Adult Liver Cells

**DOI:** 10.1155/2016/6040146

**Published:** 2016-06-15

**Authors:** Anthony Finoli, Eva Schmelzer, Patrick Over, Ian Nettleship, Joerg C. Gerlach

**Affiliations:** ^1^Department of Mechanical Engineering and Materials Science, University of Pittsburgh, Pittsburgh, PA 15261, USA; ^2^Department of Surgery, McGowan Institute for Regenerative Medicine, University of Pittsburgh, Pittsburgh, PA 15203, USA; ^3^Department of Bioengineering, McGowan Institute for Regenerative Medicine, University of Pittsburgh, Pittsburgh, PA 15203, USA

## Abstract

Liver cell culture within three-dimensional structures provides an improved culture system for various applications in basic research, pharmacological screening, and implantable or extracorporeal liver support. Biodegradable calcium-based scaffolds in such systems could enhance liver cell functionality by providing endothelial and hepatic cell support through locally elevated calcium levels, increased surface area for cell attachment, and allowing three-dimensional tissue restructuring. Open-porous hydroxyapatite scaffolds were fabricated and seeded with primary adult human liver cells, which were embedded within or without gels of extracellular matrix protein collagen-1 or hyaluronan. Metabolic functions were assessed after 5, 15, and 28 days. Longer-term cultures exhibited highest cell numbers and liver specific gene expression when cultured on hydroxyapatite scaffolds in collagen-1. Endothelial gene expression was induced in cells cultured on scaffolds without extracellular matrix proteins. Hydroxyapatite induced gene expression for cytokeratin-19 when cells were cultured in collagen-1 gel while culture in hyaluronan increased cytokeratin-19 gene expression independent of the use of scaffold in long-term culture. The implementation of hydroxyapatite composites with extracellular matrices affected liver cell cultures and cell differentiation depending on the type of matrix protein and the presence of a scaffold. The hydroxyapatite scaffolds enable scale-up of hepatic three-dimensional culture models for regenerative medicine applications.

## 1. Introduction

Culture of liver cells within three-dimensional (3D) structures provides improved* in vitro* systems for studying hepatic cell differentiation and proliferation. The unfulfilled demand for donor organs for transplantation in chronic liver disease suggests that the development of engineered tissue transplants is necessary to provide additional metabolic capacity. Regenerative medicine techniques are currently being investigated to supplement the need for transplantable tissue. Though the developments are promising, many aspects of such technologies and procedures must be examined. Chief among the unsolved problems is the need for procedures addressing the supply of human liver tissue and cells [1], most likely expanded by proliferation* in vitro* [2]. Once such a cell source has been identified, further applications could be of interest, such as extracorporeal liver support in acute liver failure [3] and pharmacologic* in vitro* screening of hepatic drug candidates [4, 5].

Early mouse liver cell cultures were introduced in monolayer by Evans et al. in the 1950s [6]. Later on, liver cell cultures in suspensions were developed [7]. With the introduction of extracellular matrices as dish coatings for monolayer cultures, the early loss of hepatic functions and viability in culture was somewhat improved. Liver relevant extracellular matrices such as laminin, various collagen types, hyaluronic acid hydrogel, and matrigel were implemented [8–13]. The use of sandwich culture [14–16], that is, the embedding of liver cells between two layers of collagen, was an important step in the enhancement of liver cell culture by mimicking the* in vivo* liver plate architecture. To realistically grow liver tissue from adult liver cells at higher densities and thus approach a more natural tissue situation, the use of a three-dimensional scaffold is advisable and a scale-up of such techniques may support the cells in cultures at a size suitable for clinical use. Such a scaffold structure must have a high porosity to facilitate cell seeding and fluid flow for mass exchange around the cells but also allow sufficient space for neoendothelialized structures, while being rigid enough to structurally support the mass of the growing tissue.

Ceramic foaming techniques have been developed to create such highly porous, permeable structures [17–19]. Biocompatible calcium phosphates, specifically hydroxyapatite, have successfully been used as scaffolds for culturing several different cell types including liver cells from both human and rat sources [20, 21]. Calcium phosphate ceramics can also be manufactured to be bioresorbable by using tricalcium phosphate or taking advantage of a high temperature decomposition of hydroxyapatite to resorbable calcium phosphate phases. In the present study, we have analyzed two different extracellular matrix proteins, collagen-1 and hyaluronic acid, in combination with previously developed porous hydroxyapatite scaffolds [19]. We investigated the influence of these conditions on longer-term cultures of human adult liver cells to create scaled-up tissue structures.

## 2. Materials and Methods

### 2.1. Cell Culture

Hydroxyapatite foam scaffolds were prepared from a 1 mm thick section of an emulsion foam as described in previous work [19]. Scaffolds were placed in polystyrene tissue culture plates (Becton Dickinson Biosciences) and were sterilized by autoclaving. Human total fresh liver cell suspensions from male donors were obtained from discarded grafts (Becton Dickinson Biosciences, Woburn, MA) and cell viability was determined by trypan blue exclusion. Cell number was counted in a Neubauer chamber. The cells were applied to the scaffolds either directly without extracellular matrix, or embedded in extracellular matrix protein. Two different gels of extracellular matrix proteins were prepared for the study. Hyaluronan gel (Glycosan, Alameda, CA) was prepared by dissolving freeze-dried hyaluronan in 1 mL of sterile water at 37°C. To decrease gelation time, a cross-linker (extralink) was added, and the prepared cell fraction was suspended in the mixture at a concentration of 1E6 cells/mL of gel. Collagen-1 gel was prepared by mixing rat-tail collagen type 1 (Becton Dickinson Biosciences) with sterile water, sterile 10x PBS, and sterile 1 M NaOH according to manufacturer's protocol. Cells were suspended at a concentration of 1E6 cells/mL of gel. To each well of a culture plate containing either scaffold or no scaffold, 250 *μ*L of the cell mixtures was added and set at 37°C within 30 minutes. Controls included also cells suspended in 250 *μ*L culture medium without addition of extracellular matrix protein. Again the concentration was 1E6 cells/mL per well. Additionally, 250 *μ*L of supplemented Williams E medium was added to each well. Williams E medium (Life Technologies, Carlsbad, CA) was supplemented with 10% fetal bovine serum, antibiotic/antimycotic mix, 2 mM glutamax (all Life Technologies), 5 *μ*g/L insulin, 10 *μ*g/L transferrin, 30 nM selenium, and 100 nM hydrocortisone (all Sigma-Aldrich, St. Louis, MO). The medium was replaced every 2 to 3 days during culture and the aspirate was saved for future protein analysis. For all experiments, cultures were kept for 5, 15, and 28 days and there were 6 biological repeats, each from a different donor.

### 2.2. Albumin Enzyme-Linked Immunosorbent Assay

A standard albumin sandwich enzyme-linked immunosorbent assay was used to measure secreted albumin in medium samples. MaxiSorp Immunoplates (Nalgene Nunc International, Penfield, NY) were absorbed with anti-human albumin antibody (Bethyl Laboratories), incubated with samples or standards (Bethyl Laboratories, Montgomery, TX), and conjugated with a goat anti-human albumin horseradish-peroxidase-conjugated antibody (Bethyl Laboratories). Tetramethylbenzidine substrate solution was incubated for 5 min, the enzymatic reaction was stopped with 2 M sulfuric acid (Fisher Scientific, Pittsburgh, PA), and absorbance was read at 450 nm with a Synergy H1 hybrid reader equipped with Gen5 software version 2.00 (Bio-Tek, Winooski, VT). To quantify albumin secretion of cells in culture at longer time points (days 18–28), a linear regression was completed on the data for each sample and for each condition; ANOVA was calculated with the null hypothesis being a slope of 0 (no secretion). A *p* value of 0.05 was considered statistically significant.

### 2.3. DNA Quantification and Gene Expression Analysis

DNA was quantified to determine cell numbers of cultures, and RNA was extracted for gene expression analyses. Total DNA and RNA were extracted from cells using the AllPrep DNA/RNA Mini Kit (Qiagen, Valencia, CA). Cells were disrupted in lysis buffer of the kit on QIAshredder columns (Qiagen). Isolated DNA was quantified using the Quant-iT dsDNA BR Assay Kit on a Qubit fluorometer and compared to a sample containing 1E6 human adult liver cells from the donor. RNA was reverse transcribed to cDNA with the High-Capacity cDNA Reverse Transcription Kit (Applied Biosystems). Real-time polymerase chain reaction (PCR) was carried out using the StepOnePlus Real-Time PCR-System equipped with StepOne Software version 2.0 (Applied Biosystems). Predesigned TaqMan probes and primer sets were obtained from Applied Biosystems and used to quantify gene expression for vWF, CK19, CYP3A4, albumin, ASMA, and beta-actin using the ddCt method. Data were normalized against beta-actin expression. Negative PCR controls included no template (water).

### 2.4. Immunocytochemistry

At each of the three time points, cell culture samples were fixed with 4% para-formaldehyde (Sigma-Aldrich). Portions of these cultures were blocked with 10% goat serum (Sigma-Aldrich) and 1% FCR block (Miltenyi Biotec, Auburn, CA) in phosphate-buffered saline and stained with diamidino-phenylindole dihydrochloride (Sigma-Aldrich) for cell nuclei and AF568-conjugated phalloidin for intracellular actin filaments (Life Technologies). To evaluate CK19 expression cells were stained with mouse anti-CK19 primary antibody (Fisher Scientific) and AF488-conjugated goat anti-mouse secondary antibody (Life Technologies). To examine endothelial cells in culture separate portions were stained with rabbit anti-vWF primary antibody (Abcam, Cambridge, MA) and AF488-conjugated goat anti-rabbit secondary antibody (Life Technologies). All stainings were analyzed by confocal microscopy using a Fluoview 1000 system (Olympus, Center Valley, PA).

## 3. Results

### 3.1. Cell Viability, Attachment, and Number

Human adult liver cell suspensions had viabilities of 65–83%.

**Figure 1:**
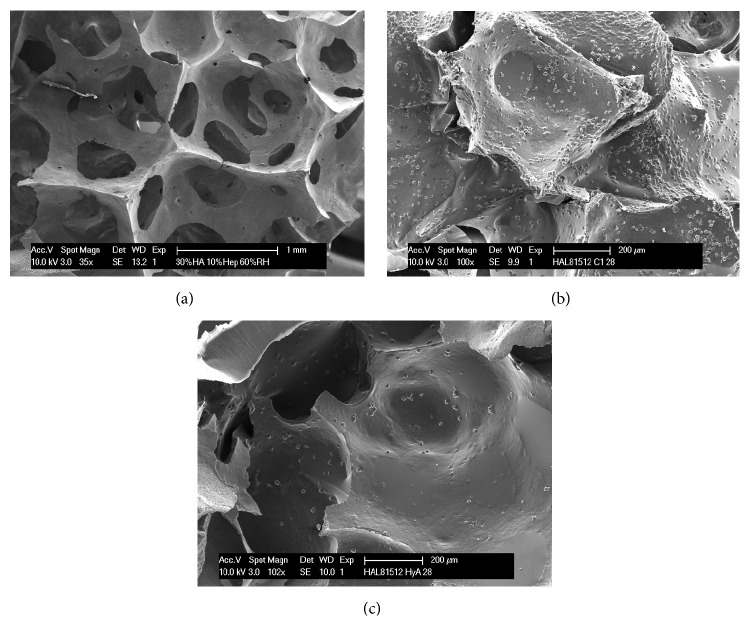
Scanning electron microscopy. Porous foamed hydroxyapatite scaffolds (a) were used for cell culture. After 28 days of culture, numerous cells attached to the scaffold could be observed in cultures with collagen-1 (b), whereas considerably less cells were attached to scaffolds in culture with hyaluronan (c).

After 28 days in culture, cells were analyzed for their attachment on hydroxyapatite scaffolds (Figure 1). In culture with collagen-1, numerous cells could be observed being attached on the scaffold (Figure 1(b)), whereas in culture with hyaluronan considerably fewer cells were attached on the scaffold (Figure 1(c)).

**Figure 2:**
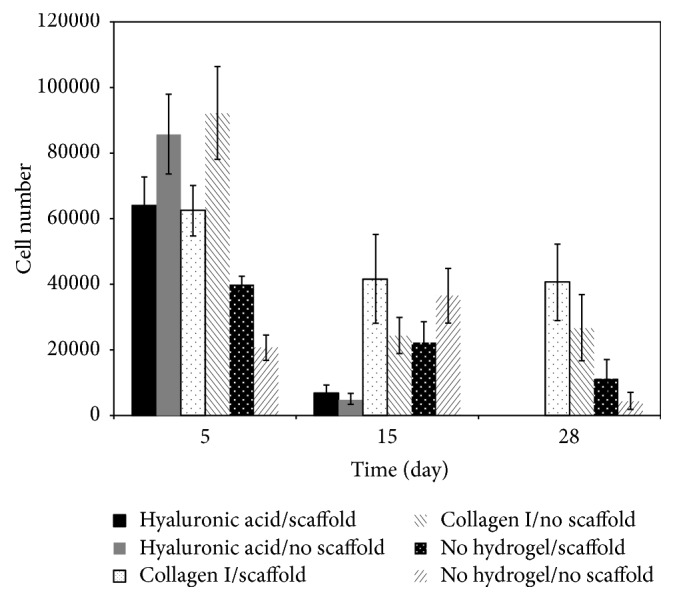
Numbers of liver cells in culture. Cells were cultured for 5, 15, and 28 days with (w) or without (wo) hydroxyapatite scaffolds embedded in hyaluronic acid gel (HyA), collagen-1 gel (C1), or no gel (NG), and cell numbers were determined by correlation with DNA concentration. Data are given as means from 6 biological repeats ± standard deviation.

We also investigated cell numbers (based on DNA correlation) in the various culture conditions after 5, 15, and 28 days of culture (Figure 2). Cell numbers in all conditions decreased after 5 days of culture when compared to initial seeding numbers. After 28 days of culture, very few cells could be detected in hyaluronan cultures, both with and without hydroxyapatite scaffold, and were significantly lower than any other condition at day 15 and day 28 (*p* < 0.05). Conversely, cells cultured in collagen-1, both with and without scaffold, showed constant cell numbers between 15 and 28 days. Cultures with the scaffolds were showing slightly higher cell numbers at 28 days than those without (40,643/well on average compared to 26,793/well).

### 3.2. Albumin ELISA

**Figure 3:**
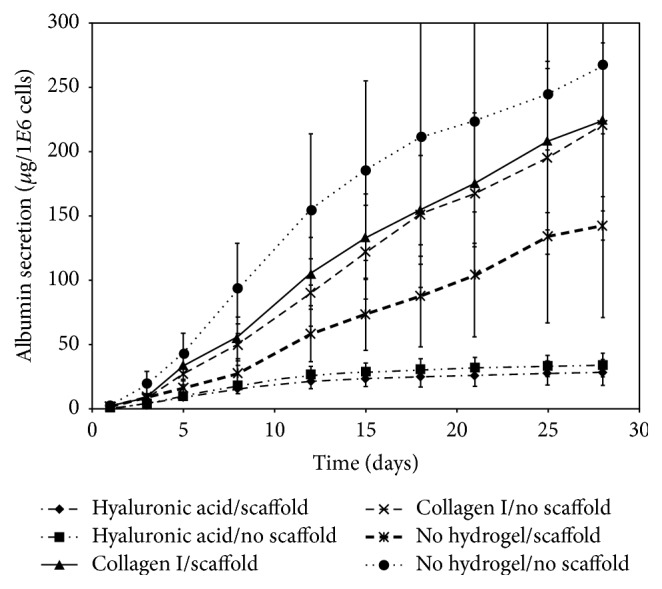
Albumin secretion of liver cell cultures. Cells were cultured for 5, 15, and 28 days with (w) or without (wo) hydroxyapatite scaffolds embedded in hyaluronic acid gel (HyA), collagen-1 gel (C1), or no gel (NG), and albumin secretion was measured by ELISA. Data are given as means from 6 biological repeats ± standard deviation.

Albumin secretion of the cultures (Figure 3) varied between samples; however, certain trends developed that were consistent between donors. Of hydrogel conditions, culture in collagen-1 showed consistently higher overall secretion than in hyaluronan. In addition, when hydroxyapatite scaffold was not present the cells ceased significantly albumin secretion after day 15. During the last ten days of culture (days 18–28) only the cells cultured on hydroxyapatite in collagen-1 showed a significant increase in albumin secretion. ANOVA linear regressions of the data demonstrated a nonzero slope in cumulative albumin secretion (*p* < 0.01) in this condition showing significant secretion in the late stages of culture (78.5 ± 40.1 (*μ*g/1E6 cells)/day). No other condition had a slope in cumulative albumin secretion significantly different from zero over the last 10 days of culture.

### 3.3. Gene Expression

Changes in expressions of genes specific for the various cell types of the liver were analyzed after different time points in culture; data are given relative to freshly isolated cells, which were set as 1. The expression of CK19, a biliary epithelium specific gene, was highly expressed in all samples (Table 1) throughout the 28-day experiment when compared to original cell suspensions. Cells cultured in collagen-1 on scaffolds had the highest expression; after 28 days cells cultured in collagen-1 with scaffold had an expression approximately 1000 times more than that of day 0 donor samples. For the other three conditions (cultures on collagen-1 without scaffold and both no-gel conditions), this expression was around 500 times that of day 0 donor cells and significantly less than cells cultured on collagen-1 with scaffold (*p* < 0.05). The expression of vWF, a mature endothelium specific marker, showed differences between samples (Table 1), with the highest expression after 15 and 28 days in cells cultured without any gel. Cells cultured without any gel on hydroxyapatite had the highest vWF expression, with expression maintained between day 15 and day 28 (*p* < 0.05). The expression of CYP3A4 (Table 1), a cytochrome P450 enzyme of mature hepatocytes, was highest in cultures with hyaluronan. Cells in hyaluronan maintained expression throughout the 28-day culture, significantly greater than all other conditions at day 28 (*p* < 0.05), and cells cultured in collagen-1 gel maintained expression through 15 days and lost expression after 28 days. Cells cultured without hydrogel downregulated expression of CYP3A4 in culture. The gene expression of albumin (Table 1), a secreted protein of mature hepatocytes, was highest in cells cultured in hyaluronan after 28 days, and always lowest in cultures without extracellular matrix addition. The expression of albumin is downregulated with the secretion of albumin, which is why cultures with high albumin secretion seen in ELISA results have lower gene expression. The downregulation of the expression of fibroblastic gene ASMA (Table 1) in most samples during culture suggests that there was no overgrowth of fibroblasts in culture.

### 3.4. Immunocytochemistry

**Figure 4:**
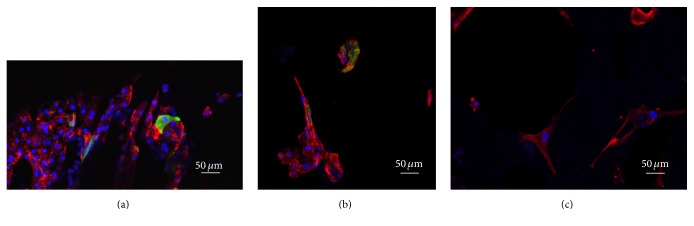
Immunocytochemistry of liver cell cultures. Liver cells were cultured for 28 days and stained, and images were taken by confocal microscopy. (a) Cells cultured in collagen-1 gel; (b) cells cultured in collagen-1 gel on hydroxyapatite scaffold; (c) cells cultured on hydroxyapatite scaffold only. Blue = DAPI, red = phalloidin, and green = CK19.

Cells cultured in collagen-1 gel, regardless of the presence of hydroxyapatite, formed hepatocyte cordlike structures similar to liver plates (Figures 4(a) and 4(b)). Cells were found in small numbers in both hyaluronic acid and the no-gel conditions without the cordlike structures seen in cells cultured in collagen-1. In addition, both collagen-1 conditions also showed many cells positive for CK19 (Figures 4(a) and 4(b)). In those samples containing hydroxyapatite, ring-like structures of CK19 positive cells were found in a few instances (Figure 4(b)), similar to bile ductular structures* in vivo*. Cells cultured on scaffolds without collagen-1 exhibited a much different morphology with cells stretching out over the surface of the ceramic (Figure 4(c)).

## 4. Discussion

The use of hydroxyapatite ceramic scaffolds should have several advantages for the scale-up of cultures. Previously [18] we reported that a heat treatment process can be used to control the relative amounts of hydroxyapatite and tricalcium phosphate in calcium phosphate scaffolds. This could open new directions in the utilization of such resorbable scaffolds for implantable constructs, as tissue implants which could initially support neovascularization and subsequently disintegrate once perfused tissue is formed. The inherent local liberation of calcium may also be of interest for creation of local calcium gradients around cells immobilized near the scaffolds surface, as calcium is thought to support endothelial structure reformation. Calcium ions contribute to the maintenance of endothelial cells and also the formation of vascularized tissue [22]. Calcium ion concentration has been shown to affect proliferation of adult rat hepatocytes directly in a tight compositional range [23]; their highest proliferation rates* in vitro* were observed at physiological concentrations of 0.4 mM while lower or higher concentrations resulted in lower proliferation rates. Biodegradable ceramic scaffolds have even been seen to influence angiogenesis in bone marrow cell cultures by creating a localized calcium rich environment [24]. The three-dimensional scaffold itself can also be used to induce endothelial cells for culture of vascularized tissue constructs by creating a surface suitable for the actin filaments of the endothelial cells to attach, as well as providing a microenvironment suitable for cell proliferation [25, 26].

Few studies have been published using ceramic structures for liver cell cultures. Ceramic plate-like structures with circular cavities were developed, and rat hepatocytes were demonstrated to attach within the cavities [27]; however, cultures were maintained only for 24 h and no liver specific functions were analyzed. The applicability of cell-seeded hydroxyapatite scaffolds for potential future clinical transplantation studies has been demonstrated by rodent transplantation studies; hydroxyapatite scaffolds seeded with immortalized mouse liver cells were successfully transplanted into the omentum and kidney of mice [21]; transplantation of hydroxyapatite disks seeded with normal rat hepatocytes intraperitoneally into Nagase analbuminemic rats significantly increased albumin secretion within the host [20].

The usefulness of extracellular matrix proteins in the improvement of hepatic cell cultures has been widely discussed in the context of cell structures for implantation or bioreactors for temporary extracorporeal use [28] (for review, see [29]). Extracellular matrix proteins not only provide mechanical stability for cell constructs but also interact directly with cells through receptors influencing their cell type specific function. We used hyaluronan and collagen-1 hydrogels for our studies on establishing culture models involving hydroxyapatite, because these extracellular proteins have been used successfully to culture human adult hepatocytes and other primary cell types [13–15, 30–32]. Of the culture conditions examined, the maintenance of cells in culture was clearly best in the collagen-1/hydroxyapatite composite showing almost two times as many cells after 28 days compared with any other condition. These findings were also supported by the other parameters examined. Although gene expression of albumin, a secreted protein of mature hepatocytes, was highest in cells cultured in hyaluronan after 28 days, actual secretion of albumin protein (as measured by ELISA) was higher in collagen-1 culture. This fact can be explained by the known feedback mechanism of albumin protein on gene expression, by which gene expression of albumin is downregulated with the secretion of albumin protein [33], which is why cultures with high albumin secretion seen in ELISA results have lower gene expression. Collagen-1 hydrogels are a common culture model for liver cells because of the abundance in liver tissue [34, 35]. This extracellular matrix provides an environment on the lowest level of organ structure similar to native liver tissue and might be expected to be the best hydrogel for supporting the culture of adult liver cells* in vitro*. When cells were cultured on porous hydroxyapatite in collagen-1 the composite structure also provided improved cell maintenance during the last two weeks of culture, compared to the negative control and also the collagen-1 sandwich cultures of primary human hepatocytes found in the literature [31]. We have shown previously that such a three-dimensional condition supported hepatic cell differentiation and proliferation in perfusion culture [32, 36]. However, it must be noted that in the model used here the static conditions did not involve medium perfusion, which was found to further enhance culture longevity [37, 38].

## Figures and Tables

**Table 1:** Gene expression of liver cell cultures.

Gene	Culture time (days)	Hyaluronic acid		Collagen I		No hydrogel	
		Scaffold	No scaffold	Scaffold	No scaffold	Scaffold	No scaffold
CK19	5	288.16	95.97	263.85	192.42	164.23	148.33
	15	453.34	202.45^*∗*^	2063.79^*∗*^	278.88	391.36	145.73
	28	873.87	791.60	1064.61	309.64	309.73	274.06

vWF	5	1.39	0.84	0.50	0.58	1.18	0.28
	15	0.078^<^	0.06^<^	0.93	1.65	4.27^*∗*^	2.64^*∗*^
	28	0.12	0.03	0.19^<^	0.34^<^	3.62	0.80^<^

CYP450 3A4	5	63.15	40.56	0.76	0.81	0.26	0.03
	15	5.35^<^	32.39	4.11	2.14	0.11	0.02
	28	41.87	39.76	0.04	0.04	0.02	0.02

Albumin	5	0.46	0.43	0.56	0.58	0.10	0.20
	15	2.03^*∗*^	0.34	0.35	0.43	0.15	0.21
	28	0.27^<^	0.42	0.16	0.12	0.08	0.12

ASMA	5	0.00	0.55	24.62	36.62	17.57	14.69
	15	0.00	10.21^*∗*^	4.06^<^	18.34	6.88	3.19^<^
	28	4.64^*∗*^	0.00^<^	2.50	0.99^<^	1.50	1.37

Cells were cultured for 5, 15, and 28 days with (w) or without (wo) hydroxyapatite scaffolds embedded in hyaluronic acid gel (HyA), collagen-1 gel (C1), or no gel (NG), and gene expression was measured by PCR for cytokeratin-19 (CK19), Von Willebrand Factor (vWF), cytochrome P450 3A4 (CYP3A4), albumin, and Alpha-Smooth Muscle Actin (ASMA). Data are given as means from 6 biological repeats, normalized against beta-actin expression, and significance (*p* < 0.05) is marked by *∗* to indicate a value significantly greater than the previous time point and < to indicate a value significantly less than the previous time point.

## References

[1] Gridelli B., Vizzini G., Pietrosi G. (2012). Efficient human fetal liver cell isolation protocol based on vascular perfusion for liver cell-based therapy and case report on cell transplantation. *Liver Transplantation*.

[2] Miki T., Ring A., Gerlach J. (2011). Hepatic differentiation of human embryonic stem cells is promoted by three-dimensional dynamic perfusion culture conditions. *Tissue Engineering Part C: Methods*.

[3] Gerlach J. C., Zeilinger K., Patzer J. F. (2008). Bioartificial liver systems: why, what, whither?. *Regenerative Medicine*.

[4] Zeilinger K., Schreiter T., Darnell M. (2011). Scaling down of a clinical three-dimensional perfusion multicompartment hollow fiber liver bioreactor developed for extracorporeal liver support to an analytical scale device useful for hepatic pharmacological in vitro studies. *Tissue Engineering—Part C: Methods*.

[5] Lubberstedt M., Muller-Vieira U., Biemel K. M. (2015). Serum-free culture of primary human hepatocytes in a miniaturized hollow-fibre membrane bioreactor for pharmacological in vitro studies. *Journal of Tissue Engineering and Regenerative Medicine*.

[6] Evans V. J., Earle W. R., Wilson E. P., Waltz H. K., Mackey C. J. (1952). The growth in vitro of massive cultures of liver cells. *Journal of the National Cancer Institute*.

[7] Gerschenson L. E., Casanello D. (1968). Metabolism of rat liver cells cultured in suspension: insulin and glucagon effects on glycogen level. *Biochemical and Biophysical Research Communications*.

[8] Hirata K., Yoshida Y., Shiramatsu K., Freeman A. E., Hayasaka H. (1983). Effects of laminin, fibronectin and type IV collagen on liver cell cultures. *Experimental Cell Biology*.

[9] Bissell D. M., Arenson D. M., Maher J. J., Roll F. J. (1987). Support of cultured hepatocytes by a laminin-rich gel. Evidence for a functionally significant subendothelial matrix in normal rat liver. *Journal of Clinical Investigation*.

[10] Brown S. E. S., Guzelian C. P., Schuetz E., Quattrochi L. C., Kleinman H. K., Guzelian P. S. (1995). Critical role of extracellular matrix on induction by phenobarbital of cytochrome P450 2B1/2 in primary cultures of adult rat hepatocytes. *Laboratory Investigation*.

[11] Block G. D., Locker J., Bowen W. C. (1996). Population expansion, clonal growth, and specific differentiation patterns in primary cultures of hepatocytes induced by HGF/SF, EGF and TGF*α* in a chemically defined (HGM) medium. *Journal of Cell Biology*.

[12] Nakajima H., Shimbara N. (1996). Functional maintenance of hepatocytes on collagen gel cultured with simple serum-free medium containing sodium selenite. *Biochemical and Biophysical Research Communications*.

[13] Turner W. S., Schmelzer E., McClelland R., Wauthier E., Chen W., Reid L. M. (2007). Human hepatoblast phenotype maintained by hyaluronan hydrogels. *Journal of Biomedical Materials Research—Part B Applied Biomaterials*.

[14] Dunn J. C. Y., Yarmush M. L., Koebe H. G., Tompkins R. G. (1989). Hepatocyte function and extracellular matrix geometry: long-term culture in a sandwich configuration. *FASEB Journal*.

[15] Dunn J. C. Y., Tompkins R. G., Yarmush M. L. (1991). Long-term in vitro function of adult hepatocytes in a collagen sandwich configuration. *Biotechnology Progress*.

[16] Dunn J. C. Y., Tompkins R. G., Yarmush M. L. (1992). Hepatocytes in collagen sandwich: evidence for transcriptional and translational regulation. *Journal of Cell Biology*.

[17] Barg S., Soltmann C., Andrade M., Koch D., Grathwohl G. (2008). Cellular ceramics by direct foaming of emulsified ceramic powder suspensions. *Journal of the American Ceramic Society*.

[18] Finoli A., McKeel D., Gerlach J., Nettleship I. (2010). Phase transformation behaviour of hydroxyapatite foams subject to heat treatment. *Biomedical Materials*.

[19] Finoli A., Ostrowski N., Schmelzer E., Nettleship I., Gerlach J. (2012). Multiscale porous ceramic scaffolds for in vitro culturing of primary human cells. *Advances in Applied Ceramics*.

[20] Higashiyama S., Noda M., Muraoka S. (2003). Transplantation of hepatocytes cultured on hydroxyapatite into Nagase analbuminemia rats. *Journal of Bioscience and Bioengineering*.

[21] Saito R., Ishii Y., Ito R. (2011). Transplantation of liver organoids in the omentum and kidney. *Artificial Organs*.

[22] Nilius B., Droogmans G. (2001). Ion channels and their functional role in vascular endothelium. *Physiological Reviews*.

[23] Eckl P. M., Whitcomb W. R., Michalopoulos G., Jirtle R. L. (1987). Effects of EGF and calcium on adult parenchymal hepatocyte proliferation. *Journal of Cellular Physiology*.

[24] Nakamura S., Matsumoto T., Sasaki J.-I. (2010). Effect of calcium ion concentrations on osteogenic differentiation and hematopoietic stem cell niche-related protein expression in osteoblasts. *Tissue Engineering—Part A*.

[25] Santos M. I., Reis R. L. (2010). Vascularization in bone tissue engineering: physiology, current strategies, major hurdles and future challenges. *Macromolecular Bioscience*.

[26] Nguyen L. H., Annabi N., Nikkhah M. (2012). Vascularized bone tissue engineering: approaches for potential improvement. *Tissue Engineering Part B: Reviews*.

[27] Petronis S., Eckert K.-L., Gold J., Wintermantel E. (2001). Microstructuring ceramic scaffolds for hepatocyte cell culture. *Journal of Materials Science: Materials in Medicine*.

[28] Sauer I. M., Kardassis D., Zeillinger K. (2003). Clinical extracorporeal hybrid liver support—phase I study with primary porcine liver cells. *Xenotransplantation*.

[29] Drury J. L., Mooney D. J. (2003). Hydrogels for tissue engineering: scaffold design variables and applications. *Biomaterials*.

[30] Lee K. Y., Mooney D. J. (2001). Hydrogels for tissue engineering. *Chemical Reviews*.

[31] Weiss T. S., Jahn B., Cetto M., Jauch K.-W., Thasler W. E. (2002). Collagen sandwich culture affects intracellular polyamine levels of human hepatocytes. *Cell Proliferation*.

[32] Schmelzer E., Triolo F., Turner M. E. (2010). Three-dimensional perfusion bioreactor culture supports differentiation of human fetal liver cells. *Tissue Engineering—Part A*.

[33] Pietrangelo A., Panduro A., Chowdhury J. R., Shafritz D. A. (1992). Albumin gene expression is down-regulated by albumin or macromolecule infusion in the rat. *Journal of Clinical Investigation*.

[34] Martinez-Hernandez A. (1984). The hepatic extracellular matrix. I. Electron immunohistochemical studies in normal rat liver. *Laboratory Investigation*.

[35] Martinez-Hernandez A., Amenta P. S. (1993). *Morphology, Localization, and Origin of the Hepatic Extracellular Matrix*.

[36] Ring A., Gerlach J., Peters G. (2010). Hepatic maturation of human fetal hepatocytes in four-compartment three-dimensional perfusion culture. *Tissue Engineering—Part C: Methods*.

[37] Gerlach J. C., Mutig K., Sauer I. M. (2003). Use of primary human liver cells originating from discarded grafts in a bioreactor for liver support therapy and the prospects of culturing adult liver stem cells in bioreactors: a morphologic study. *Transplantation*.

[38] Zeilinger K., Holland G., Sauer I. M. (2004). Time course of primary liver cell reorganization in three-dimensional high-density bioreactors for extracorporeal liver support: an immunohistochemical and ultrastructural study. *Tissue Engineering*.

